# IGF1R deficiency attenuates acute inflammatory response in a bleomycin-induced lung injury mouse model

**DOI:** 10.1038/s41598-017-04561-4

**Published:** 2017-06-27

**Authors:** Sergio Piñeiro-Hermida, Icíar P. López, Elvira Alfaro-Arnedo, Raquel Torrens, María Iñiguez, Lydia Alvarez-Erviti, Carlos Ruíz-Martínez, José G. Pichel

**Affiliations:** 1 Lung Cancer and Respiratory Diseases Unit, Centro de Investigación Biomédica de La Rioja (CIBIR), Fundación Rioja Salud, Logroño, Spain; 2 Genomics Core Facility, Centro de Investigación Biomédica de La Rioja (CIBIR), Fundación Rioja Salud, Logroño, Spain; 3 Molecular Neurobiology Unit, Centro de Investigación Biomédica de la Rioja (CIBIR), Fundación Rioja Salud, Logroño, Spain; 4 Pneumology Service, Hospital San Pedro, Logroño, Spain

## Abstract

IGF1R (Insulin-like Growth Factor 1 Receptor) is a tyrosine kinase with pleiotropic cellular functions. IGF activity maintains human lung homeostasis and is implicated in pulmonary diseases such as cancer, ARDS, COPD, asthma and fibrosis. Here we report that lung transcriptome analysis in mice with a postnatally-induced *Igf1r* gene deletion showed differentially expressed genes with potentially protective roles related to epigenetics, redox and oxidative stress. After bleomycin-induced lung injury, IGF1R-deficient mice demonstrated improved survival within a week. Three days post injury, IGF1R-deficient lungs displayed changes in expression of IGF system-related genes and reduced vascular fragility and permeability. Mutant lungs presented reduced inflamed area, down-regulation of pro-inflammatory markers and up-regulation of resolution indicators. Decreased inflammatory cell presence in BALF was reflected in diminished lung infiltration mainly affecting neutrophils, also corroborated by reduced neutrophil numbers in bone marrow, as well as reduced lymphocyte and alveolar macrophage counts. Additionally, increased SFTPC expression together with hindered HIF1A expression and augmented levels of *Gpx8* indicate that IGF1R deficiency protects against alveolar damage. These findings identify IGF1R as an important player in murine acute lung inflammation, suggesting that targeting IGF1R may counteract the inflammatory component of many lung diseases.

## Introduction

Inflammation is a relevant component of many lung diseases including ARDS, COPD, asthma, cancer, fibrosis and pneumonia^1–5^. Early inflammatory stages of lung injury have been experimentally studied using the bleomycin (BLM) mouse model because of its low complexity and high reproducibility. BLM treatment mediates the generation of reactive oxygen species and subsequent DNA damage in the lung^6–8^. In mice, BLM induces alveolar damage and pulmonary inflammation with an initial elevation of cytokines such as IL1B, TNF and IL6, which lead to acute lung injury within a week^6, 8^. These pro-inflammatory mediators, released by alveolar macrophages, up-regulate the expression of cell adhesion molecules and stimulate the endothelium to produce chemokines, which in turn promote migration of neutrophils into alveolar spaces. Activation of both neutrophils and macrophages further induces the release of additional pro-inflammatory mediators and reactive oxygen species, resulting in apoptosis or necrosis of alveolar type 1 cells, and consequently increased permeability of the alveolar-capillary barrier, lung edema and inactivation of surfactant production^5, 9, 10^.

The insulin-like growth factor 1 receptor (IGF1R) is a ubiquitously expressed membrane-bound tyrosine kinase that mediates the positive effects of its ligands, IGF1 and IGF2, to control a number of essential biological outcomes. IGF activity and availability are modulated by six high-affinity IGF binding proteins (IGFBPs). IGF1R signaling primarily results in activation of the MAP Kinase and PI3 Kinase/Akt downstream pathways that modulate multiple cellular functions at the endocrine, paracrine and autocrine levels such as growth, proliferation, differentiation, survival, adhesion and migration^11, 12^. IGF activity was extensively reported in maintaining human lung homeostasis, as it is involved in relevant respiratory diseases including cancer, COPD, fibrosis and ARDS^13–16^.

IGF1R is highly relevant in the murine lung, displaying the highest activation levels of any organ upon challenge with IGF1^17^. Additionally, epithelial-specific *Igf1r* deficient mice showed disturbed airway epithelial differentiation after naphthalene-induced club cell injury^18^, and mice with compromised IGF1R signalling displayed oxidative stress resistance^19, 20^. Moreover, ablation of the macrophage IGF1-IGF1R axis inhibits the NLRP3 inflammasome, a protein complex that is activated in response to BLM-induced acute lung injury, which indicates that IGF1R plays an important role in initiation of the inflammatory process^21, 22^. On this basis, we aimed to study the implications of IGF1R on the inflammatory process that occurs during BLM-induced acute lung injury. For this purpose we used the recently characterized *Igf1r* conditional mutant mice *UBC-CreERT2*; *Igf1r* ^*fl*/*fl*^ (*CreERT2*)^23^. In this study, lung transcriptome analysis of *CreERT2* mice showed differential expression of genes that could serve a protective role in the lung, and it was also demonstrated that IGF1R deficiency confers resistance to BLM-mediated acute lung injury by counteracting the pulmonary inflammatory response. These results contribute toward a better understanding of the importance of IGF1R as a potential target for future therapeutic approaches in lung diseases with an inflammatory component.

## Results

### Postnatal IGF1R deficiency in *CreERT2* mice causes a general inhibition of differentially expressed genes in the prepubertal lung

**Figure 1:**
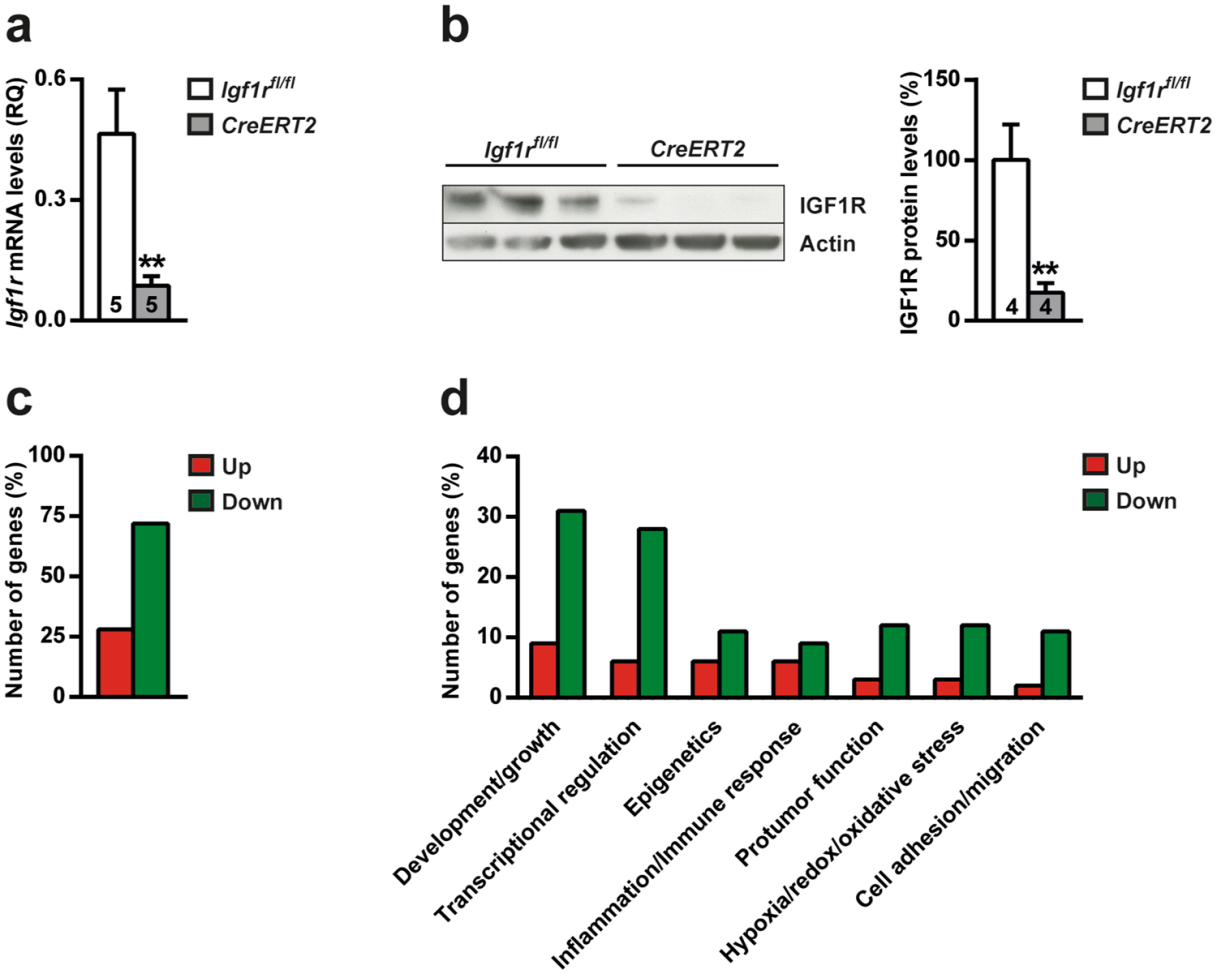
Decreased IGF1R expression levels and changes in the lung transcriptome of prepubertal IGF1R*-*deficient mice. (**a**) *Igf1r* mRNA expression levels and (**b**) representative Western blots for IGF1R and graphical representation of densitometric measurements of band intensities (percentage) normalized to beta-Actin levels and quantified in eight-week-old *UBC-CreERT2*; *Igf1r* ^*fl*/*fl*^ (*CreERT2*) mice with respect to controls (*Igf1r* ^*fl*/*fl*^). (**c**) Number of differentially expressed genes (percentage) found with significant changes (FDR < 0.1) in the lungs of *UBC-CreERT2*; *Igf1r* ^*fl*/*fl*^ (*CreERT2*) mice (n = 3), and (**d**) representation of the percentage of *Igf1r*-transcriptionally regulated genes involved in reported biological functions. Bars are color-coded in red and green for up- and down-regulated genes (respectively). Numbers within graphic bars indicate the number of mice analyzed and data are expressed as mean ± SEM. **p < 0.01 (Mann-Whitney U test). Up, up-regulated; Down, down-regulated.

To study the effect of IGF1R deficiency in the postnatal mouse lung, *CreERT2* mice were treated with tamoxifen at four weeks of age to induce *Igf1r* gene deletion^23^. Quantitative real-time PCR (qRT-PCR) and Western blot analyses on lung extracts of eight-week-old *CreERT2* tamoxifen-treated mice verified efficient depletion of IGF1R expression at the RNA and protein levels (81% and 82%, respectively), when compared to their control littermates (*Igf1r* ^*fl*/*fl*^) (Fig. 1a,b). To determine the impact of *Igf1r* deficiency on global lung RNA gene expression, RNA-Seq was performed. After bioinformatics analyses comparing *CreERT2 vs*. *Igf1r* ^*fl*/*fl*^ lung mRNA expression profiles, significant changes in gene expression were found (data submitted to Gene Expression Omnibus, accession number GSE88908). Establishing a False Discovery Rate (FDR) <0.1, 65 differentially expressed genes were identified. 18 genes were up-regulated (28%) and 47 were down-regulated (72%) (Fig. 1c and Supplementary Table S1). The most significantly affected biological functions based on GO and Keyword annotations, as well as published reports, are shown in Fig. 1d, and genes assigned to an extended list of these functions are displayed in Supplementary Table S2. Interestingly, the majority of genes in all categories were down-regulated. Most of them fall into three major categories: development/growth (*Mki67* and *Rps7*, up-regulated; *Notch3*, *Foxo1*, *Epas1* and *Tgfbr3*, down-regulated), transcriptional regulation and epigenetics (*Top2a*, *Hist1h1d* and *Hist1h2bb*, up-regulated; *Crebbp*, *Ep300*, *Polr2a*, *Zbtb34*, *Zbtb16*, *Zfp518b* and *Zfhx3*, down-regulated). Additional relevant biological functions of these genes include inflammation and immune response, followed by protumor function, hypoxia/redox and oxidative stress, as well as cell adhesion and migration (Supplementary Table S2). The top ten differentially expressed genes and their major biological functions are listed in Table 1. As expected, *Igf1r* is the most down-regulated gene, followed by *Srrm2* (involved in pre-mRNA splicing), *Nr1d2* (transcriptional regulator) and *Epas1* (endothelial barrier integrity). The most up-regulated genes are *Gpx8* and *Cyp1a1*, both implicated in anti-oxidative stress, followed by *Gnptg* (lysosome transport), *Ppp1r2-ps4* (pseudogene) and *Saa3* and *Spon2*, both involved in the immune response.

**Table 1:** Top 10 differentially expressed genes in the lung of *UBC-CreERT2*; *Igf1r* ^*fl*/*fl*^ mutant mice, and their assigned main functions.

Accession No.	Gene name	Description	FDR	Main function
**Up-regulated**				
NM_027127.2	*Gpx8*	Glutathione peroxidase 8	5.08 E-11	Antioxidative stress
NM_009992.4	*Cyp1a1*	Cytochrome P450, family 1, subfamily a, polypeptide 1	1.51 E-10	Antioxidative stress
NM_172529.3	*Gnptg*	N-acetylglucosamine-1-phosphotransferase, gamma subunit	1.10 E-08	Lysosome transport
AK015709	*Ppp1r2-ps4*	Protein phosphatase 1, regulatory (inhibitor) subunit 2, pseudogene 4	3.69 E-05	Unknown
NM_011315.3	*Saa3*	Serum amyloid A 3	7.26 E-05	Immune cell response
NM_133903.3	*Spon2*	Spondin 2, extracellular matrix protein	3.20 E-4	Immune cell response
**Down-regulated**				
NM_010513.2	*Igf1r*	Insulin-like growth factor I receptor	6.91 E-11	Cell growth and survival
NM_175229.3	*Srrm2*	Serine/arginine repetitive matrix 2	2.90 E-06	Pre-mRNA splicing
NM_011584.4	*Nr1d2*	Nuclear receptor subfamily 1, group D, member 2	2.65 E-05	Transcriptional regulation
NM_010137.3	*Epas1*	Endothelial PAS domain protein 1	8.78 E-05	Endothelial barrier integrity

### IGF1R deficiency improves mouse survival and alters IGF system gene expression in early stages after BLM-mediated pulmonary injury

**Figure 2:**
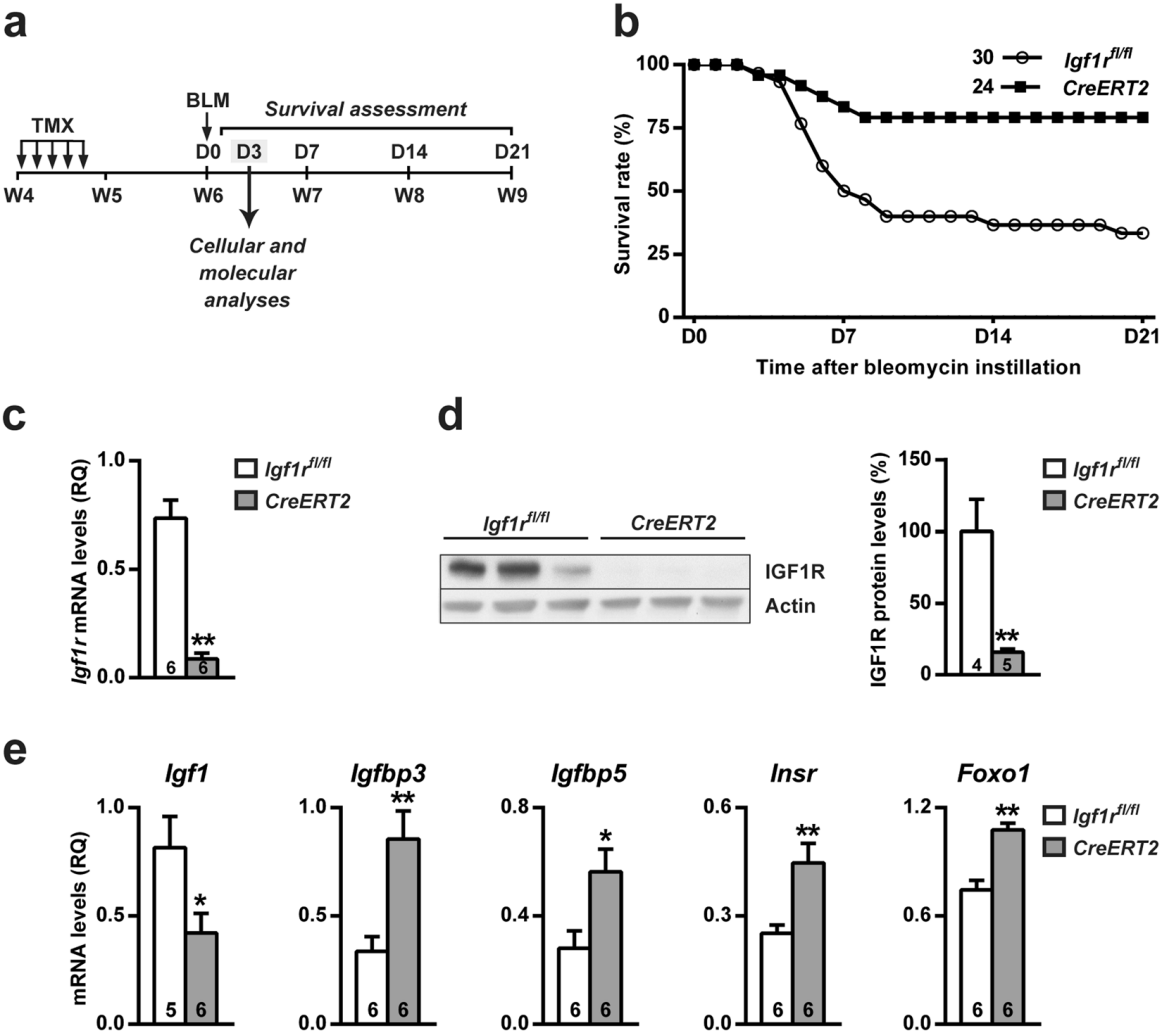
Establishment of the BLM-mediated acute lung injury model, and improved survival, reduced expression of IGF1R as well as changes in mRNA expression of IGF system genes in IGF1R-deficient mice. (**a**) Tamoxifen (TMX) was administered daily for five consecutive days to four-week-old *UBC-CreERT2*; *Igf1r* ^*fl*/*fl*^ (*CreERT2*) mice to induce a postnatal *Igf1r* gene conditional deletion as previously described using *Igf1r* ^*fl*/*fl*^ mice as experimental controls^23^. Six-week-old mice were intra-tracheally instilled with 2.5 µl/g BLM (2 U/ml) or saline using a ketamine-xylazine anesthetic combination. Cellular and molecular analyses were assessed on day (D) 3, based on survival curves. (**b**) Survival rates after BLM challenge determined over a follow-up period of 21 days in *UBC-CreERT2*; *Igf1r* ^*fl*/*fl*^ (*CreERT2*) (n = 24) and *Igf1r* ^*fl*/*fl*^ mice (n = 30). Data are expressed as the percentage of mice alive at each time point. (**c**) *Igf1r* mRNA expression levels, (**d**) representative Western blots for IGF1R and graphical representation of densitometric measurements of band intensities (percentage) normalized to beta-Actin levels, and (**e**) mRNA expression of IGF system related genes (*Igf1*, *Igfbp3*, *Igfbp5*, *Insr and Foxo1*) in lungs of *UBC-CreERT2*; *Igf1r* ^*fl*/*fl*^ (*CreERT2*) *vs*. *Igf1r* ^*fl*/*fl*^ mice at D3 post-intratracheal instillation. Numbers within graphic bars indicate the number of mice analyzed and data are expressed as mean ± SEM. *p < 0.05; **p < 0.01 (Mann-Whitney U test). BLM, bleomycin.

To further analyze how IGF1R affects lung homeostasis, *CreERT2* mice were treated with BLM to induce lung damage at six weeks of age (D0), and their survival was followed until D21 (Fig. 2a,b). The percentage of survivors after BLM challenge was significantly higher in *CreERT2* mice (79%) than in *Igf1r* ^*fl*/*fl*^ mice (33%), without gender differences. Interestingly, mortality predominantly affected mice within the first week of treatment, beginning at D3 (Fig. 2b). qRT-PCR and Western blot analyses on lung extracts at D3 verified IGF1R reduced mRNA (88%) and protein (84%) levels in *CreERT2* mice (Fig. 2c,d). In our search for possible compensatory effects on IGF system gene expression, we determined the mRNA levels of *Igf1*, *Igfbp3*, *Igfbp5* and *Insr*, in addition to the IGF/Ins transcription factor-signaling mediator *Foxo1*, by qRT-PCR. *Igf1* levels were found to be significantly diminished in *CreERT2* lungs but conversely, *Igfbp3*, *Igfbp5*, *Insr* and *Foxo1* levels were increased (Fig. 2e).

### IGF1R depletion protects against lung vascular fragility and permeability, and reduces inflammatory cell presence in BALF after BLM treatment

**Figure 3:**
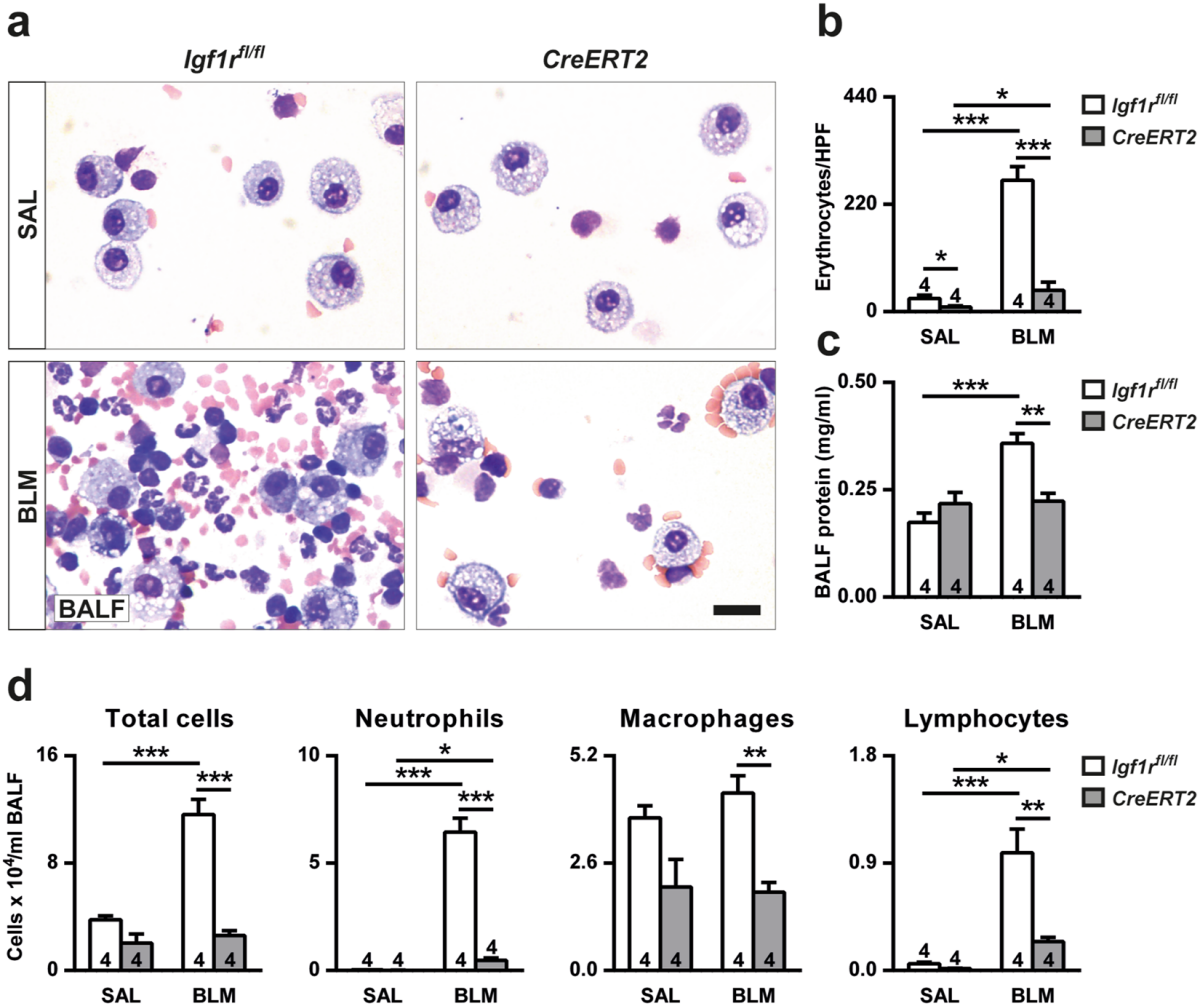
Reduced vascular fragility and lung permeability, and diminished leukocyte presence in BALF of IGF1R-deficient mice after BLM treatment. (**a**) Representative cytospin images, (**b**) quantification of number of erythrocytes per HPF, (**c**) total protein concentration and, (**d**) total and differential cell counts performed on cytospin preparations of BALF from saline- or BLM-treated *UBC-CreERT2*; *Igf1r* ^*fl*/*fl*^ (*CreERT2*) *vs*. *Igf1r* ^*fl*/*fl*^ mice at D3. Scale bar: 20 µm. Numbers within graphic bars indicate the number of mice analyzed and data are expressed as mean ± SEM. *p < 0.05; **p < 0.01 ***p < 0.001 (Dunn Sidak multiple comparison test). SAL, saline; BLM, bleomycin; BALF, bronchoalveolar lavage fluid; HPF, high-power field.

Since BLM causes an acute increase in total cells and protein concentration in bronchioalveolar lavage fluid (BALF)^6^, BALFs from saline- (SAL) and BLM-treated mice from both genotypes were analyzed at D3 (Fig. 3a). To evaluate lung vascular fragility and permeability, the presence of erythrocytes and the protein concentration in BALF were quantified. Interestingly, the increased erythrocyte presence (10-fold) found in BALF of *Igf1r* ^*fl*/*fl*^ from BLM compared to SAL-treated mice, was not as pronounced (4-fold) in *CreERT2* mice. It is important to mention that erythrocyte counts from SAL-treated *CreERT2* mice were significantly reduced (3-fold) with respect to *Igf1r* ^*fl*/*fl*^, and were even more accentuated (6-fold) after BLM challenge (Fig. 3b). Only *Igf1r* ^*fl*/*fl*^ BALF protein levels were found to be increased (2-fold) when comparing SAL- to BLM-treated mice, whereas in *CreERT2* mice, protein levels remained unchanged after BLM treatment (Fig. 3c). In parallel, total and differential cell counts for neutrophils, macrophages and lymphocytes were severely attenuated in BALF from BLM-challenged *CreERT2* lungs with respect to their SAL-treated controls (Fig. 3d). Additionally, differential cell counts were also calculated as a proportion with respect to total absolute cell numbers, and expressed as percentages (Supplementary Table S3). Although BLM-treated *Igf1r* ^*fl*/*fl*^ mice demonstrated a significant increment in BALF total cells (3-fold) compared to SAL-treated mice, *CreERT2* mice did not show such an increase. Differential cell counts for neutrophils, macrophages and lymphocytes in BLM-challenged *Igf1r* ^*fl*/*fl*^ mice exhibited the same marked increase. Furthermore, total and differential cell counts in BALF of IGF1R-deficient mice showed a severe attenuation with respect to *Igf1r* ^*fl*/*fl*^ (Fig. 3d).

### IGF1R deficiency reduces proliferation and attenuates acute lung inflammation and bone marrow neutrophilopoiesis after BLM-challenge

**Figure 4:**
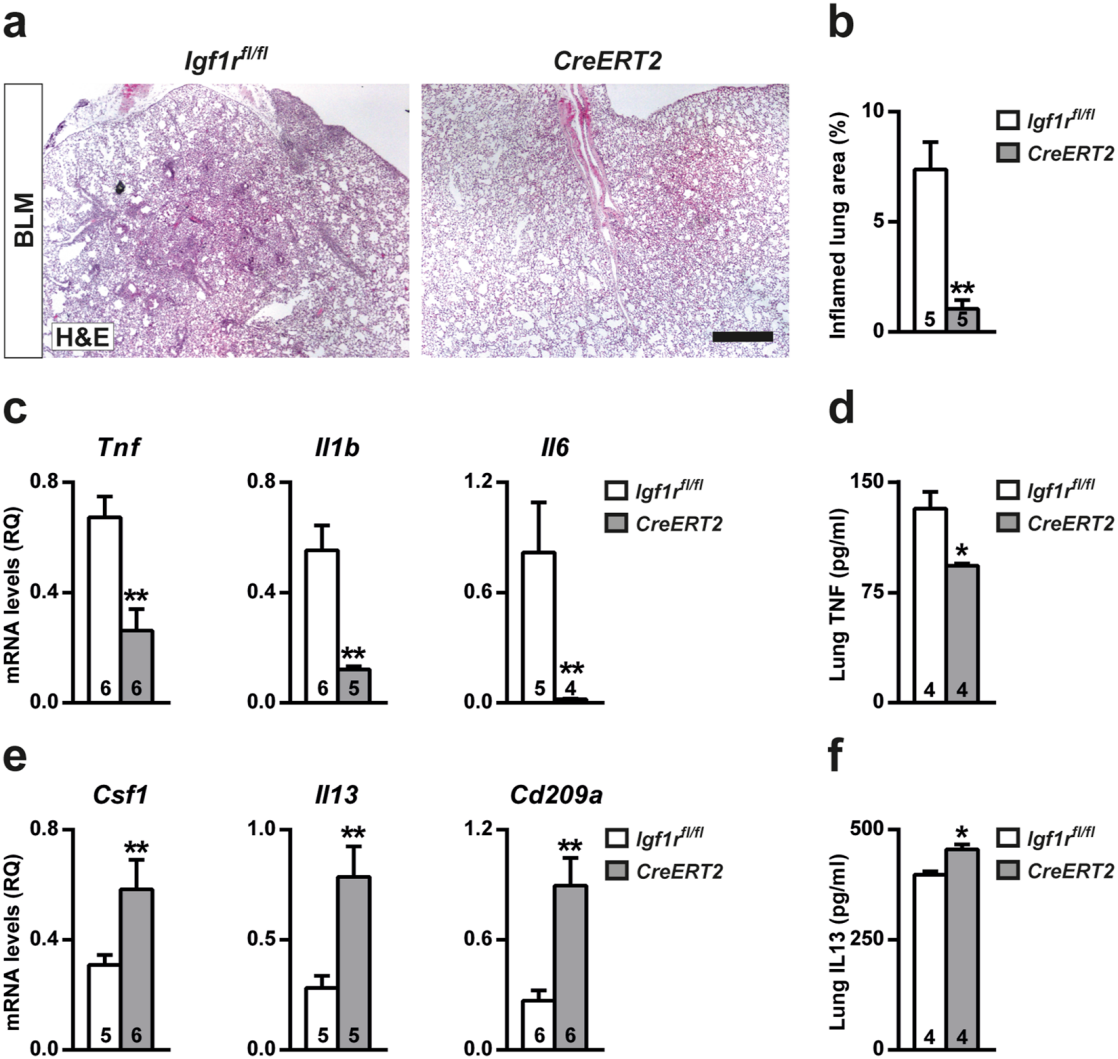
Decreased inflammation and increased resolution marker levels in IGF1R-deficient lungs following BLM treatment. (**a**) Representative images of H&E stained inflamed lung sections (scale bar: 0.5 mm), (**b**) quantification of inflamed lung area to total lung surface, (**c**,**e**) lung tissue mRNA expression of pro-inflammatory (*Tnf*, *Il1b* and Il6) and injury resolution phase (*Il13*, *Csf1* and *Cd209a*) markers, and (**d**,**f**) TNF and IL13 levels in lung homogenate from BLM-treated *UBC-CreERT2*; *Igf1r* ^*fl*/*fl*^ (*CreERT2*) mice at D3. Numbers within graphic bars indicate the number of mice analyzed and data are expressed as mean ± SEM. *p < 0.05; **p < 0.01 (Mann-Whitney U test). BLM, bleomycin.

**Figure 5:**
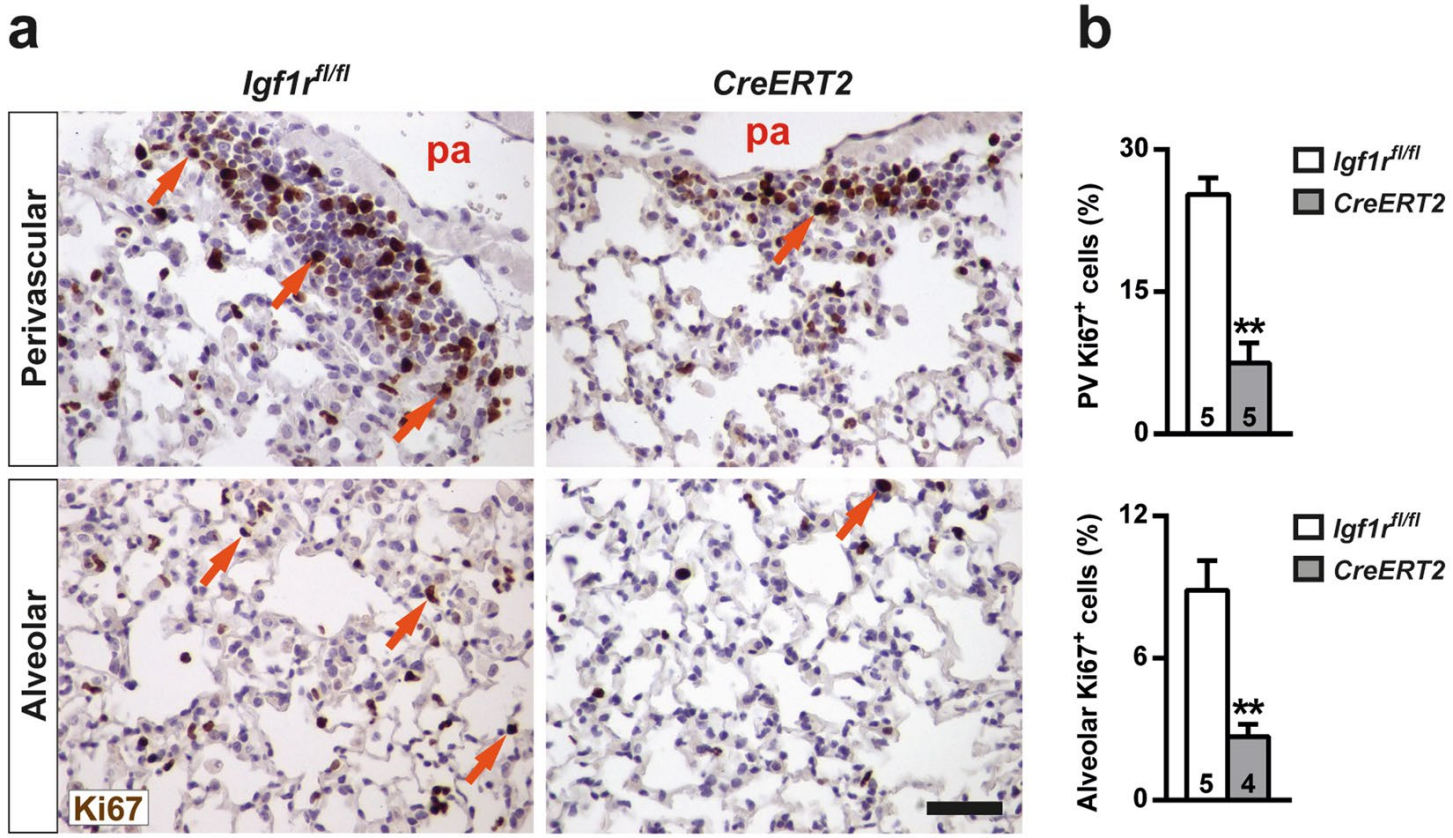
Diminished cell proliferation in IGF1R-deficient lungs after BLM challenge. (**a**) Representative images of Ki67 immuno-staining (in brown, orange arrows) under the pulmonary artery (top) and in the alveolar parenchyma (bottom), and (**b**) quantification of cell proliferation rates (Ki67^+^) in BLM treated *UBC-CreERT2*; *Igf1r* ^*fl*/*fl*^ (*CreERT2*) *vs*. *Igf1r* ^*fl*/*fl*^ lungs at D3 (scale bar: 20 μm). Numbers within graphic bars indicate the number of mice analyzed and data are expressed as mean ± SEM. **p < 0.01 (Mann-Whitney U test). PV, perivascular; pa, pulmonary artery.

**Figure 6:**
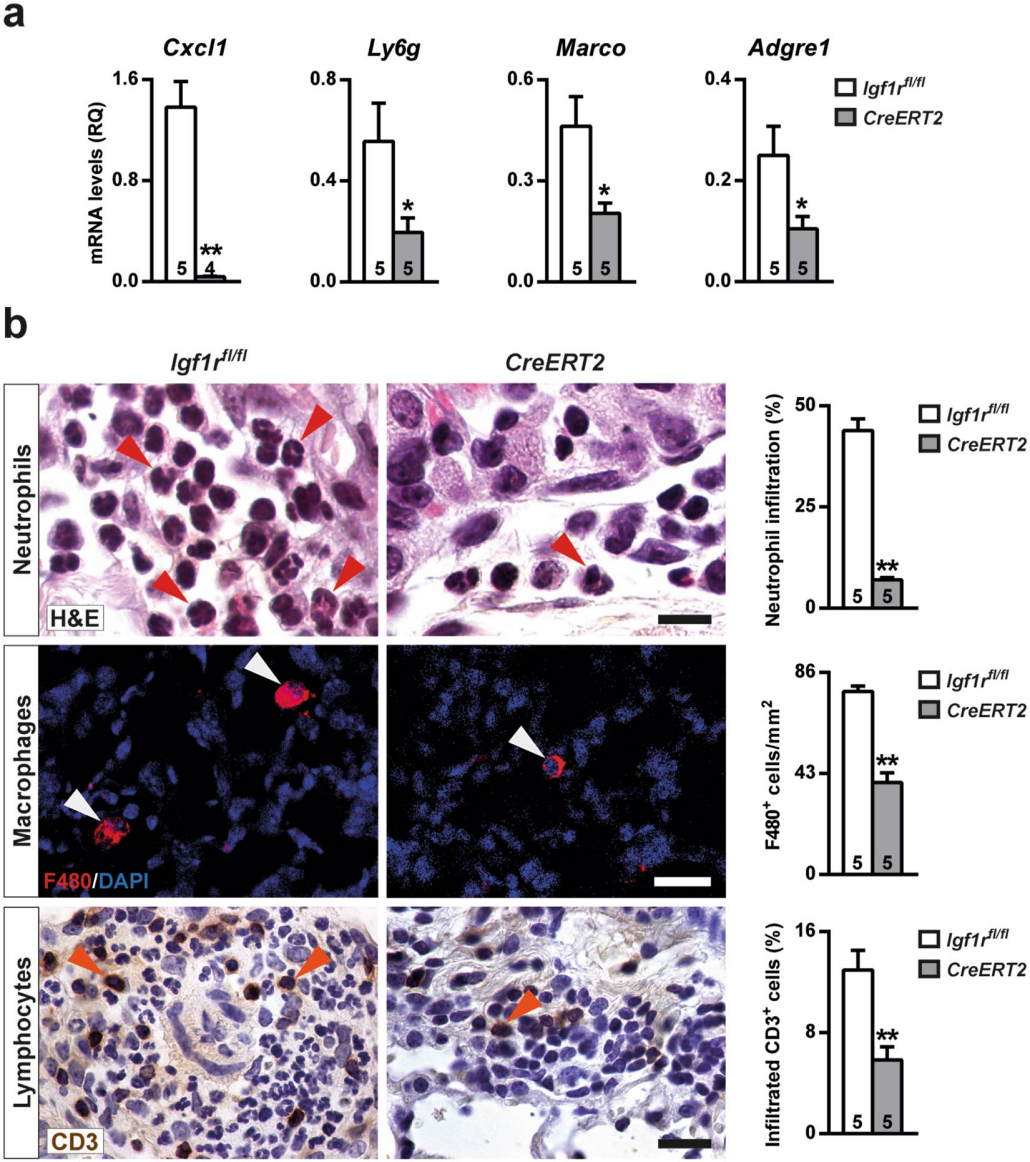
Reduced inflammatory cell infiltration in IGF1R-deficient lungs after BLM treatment. (**a**) Lung mRNA expression of neutrophil chemotaxis (*Cxcl1*), neutrophil (*Ly6g*) and macrophage (*Adgre1* and *Marco*) markers and (**b**) representative H&E, as well as F4/80 and CD3 immunostained sections, and quantification of neutrophils (red arrowheads, upper panels), alveolar macrophages (white arrowheads, middle panels) and lymphocytes (orange arrowheads, bottom panels) in BLM treated *UBC-CreERT2*; *Igf1r* ^*fl*/*fl*^ (*CreERT2*) *vs*. *Igf1r* ^*fl*/*fl*^ lungs at D3. Scale bars: 10 μm (upper panels) and 20 μm (middle and bottom panels). Numbers within graphic bars indicate the number of mice analyzed and data are expressed as mean ± SEM. *p < 0.05; **p < 0.01 (Mann-Whitney U test).

**Figure 7:**
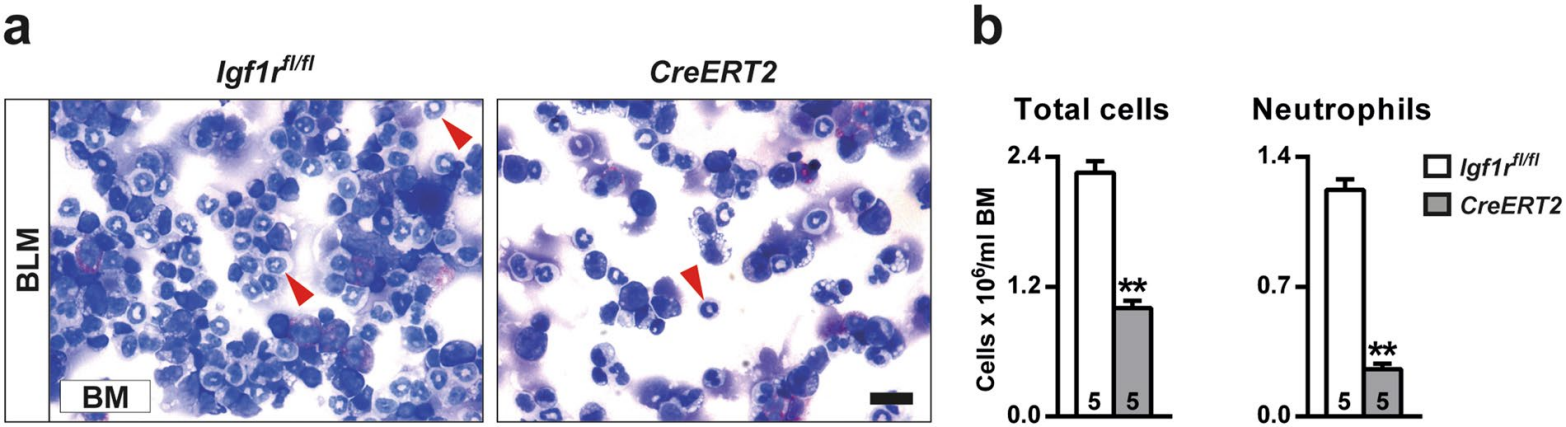
Reduced total and neutrophil counts in bone marrow from IGF1R-deficient mice. (**a**,**b**) Representative images, and total and neutrophil (red arrowheads) counts in bone marrow cytospin preparations from BLM-treated *UBC-CreERT2*; *Igf1r* ^*fl*/*fl*^ (*CreERT2*) *vs*. *Igf1r* ^*fl*/*fl*^ mice at D3. Scale bar: 20 µm. Numbers within graphic bars indicate the number of mice analyzed and data are expressed as mean ± SEM. **p < 0.01 (Mann-Whitney U test). BLM, bleomycin; BM, bone marrow.

Inflamed lung areas were measured at D3, and were found to be markedly reduced in BLM-treated *Cre-ERT2* lungs (7-fold) (Fig. 4a,b). To verify and further investigate the mechanism by which IGF1R deficiency blocks inflammatory cell recruitment to the lung, mRNA expression analysis of different inflammatory markers was performed. Pro-inflammatory cytokines *Tnf*, *Il1b* and *Il6* were found to be significantly down-regulated in *CreERT2* lungs (Fig. 4c), and the reduction of TNF protein levels was confirmed by ELISA (Fig. 4d). Conversely, mRNA expression levels of the injury resolution-phase markers *Csf1*, *Il13* and *Cd209a* were significantly increased (Fig. 4e). Despite clearly increased *Il13* mRNA levels, protein levels were found to be only slightly increased (Fig. 4f). Cell proliferation evaluated in lung perivascular areas and in the alveolar parenchyma by Ki67 immuno-staining on D3 was found to be substantially reduced in *CreERT2* mice (3-fold in both cases) (Fig. 5a,b). As an indirect measure of inflammatory cell presence in the lung, mRNA levels of neutrophil chemotaxis (*Cxcl1*), neutrophil (*Ly6g*) and macrophage (*Marco* and *Adgre1*) markers were determined. *Cxcl1* levels were greatly reduced in IGF1R-deficient lungs, and *Ly6g*, *Marco* and *Adgre1*markers also showed a significant reduction (Fig. 6a). Neutrophilic infiltration found in perivascular areas was 44% and 7% of total infiltrates for *Igf1r* ^*fl*/*fl*^ and *CreERT2* BLM-challenged lungs, respectively (Fig. 6b, upper panel). F4/80 immunostaining revealed a significant decrease for both alveolar macrophage numbers (50%), and volumes (360.1 ± 17.1 μm^3^ *vs*. 78.1 ± 5.3 μm^3^; *p* = 0.009) (Fig. 6b, middle panel). In view of the reduced presence of BALF lymphocytes in mutant mice, infiltration into the lung was also assessed by immunostaining for CD3. Accordingly, lymphocyte counts were also diminished (45%) in *CreERT2* lungs (Fig. 6b, bottom panel). To verify reduced neutrophil infiltration in IGF1R-deficient mice, total and neutrophil counts were performed in bone marrow cytospins obtained from mice of both genotypes after BLM treatment. As expected, total cells and neutrophils were found to be diminished in *CreERT2* mice with respect to *Igf1r* ^*fl*/*fl*^ BLM-challenged lungs (2- and 5-fold, respectively) (Fig. 7a,b).

### IGF1R deficiency reduces alveolar damage and HIF1A expression in BLM-challenged lungs

**Figure 8:**
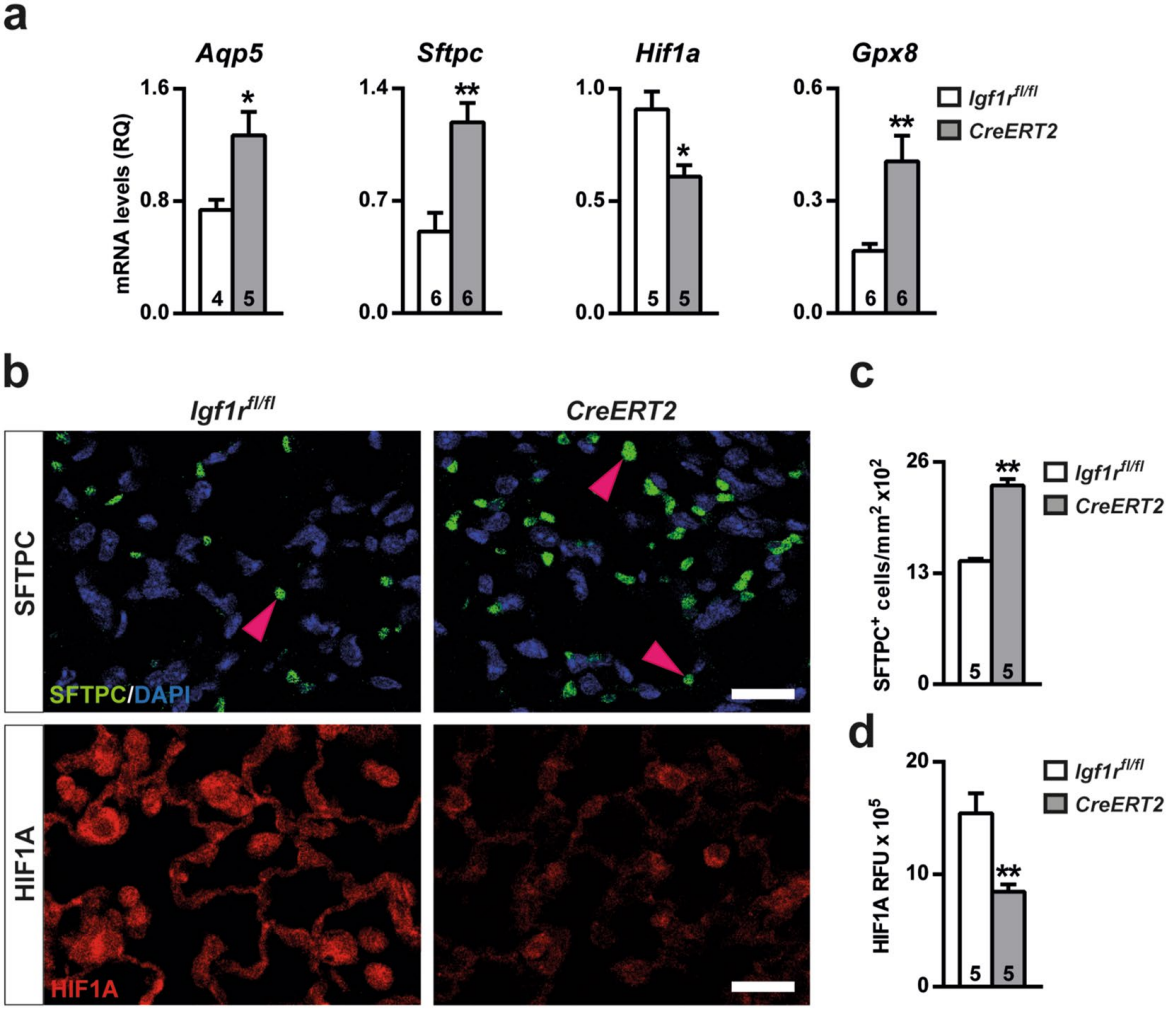
Reduced alveolar damage and HIFA expression in BLM-challenged lungs of IGF1R-deficient mice. (**a**) Changes in mRNA expression of alveolar (*Aqp5 and Sftpc*), response to hypoxia (*Hif1a*), and antioxidative stress (*Gpx8*) markers, (**b**) representative SFTPC (pink arrowheads) and HIF1A immunostained sections (upper and bottom panels, respectively), (**c**) number of SFTPC positive cells per unit area of lung tissue, and (**d**) quantification of HIF1A relative fluorescence intensity in lungs of *UBC-CreERT2*; *Igf1r* ^*fl*/*fl*^ (*CreERT2*) and *Igf1r* ^*fl*/*fl*^ mice at D3 after BLM treatment. Scale bars: 20 µm. Numbers within graphic bars indicate the number of mice analyzed and data are expressed as mean ± SEM. *p < 0.05; **p < 0.01 (Mann-Whitney U test). RFU, relative fluorescence units.

To determine the effect of IGF1R depletion on alveolar damage after BLM challenge, alveolar epithelial cell type-specific markers were quantified by qRT-PCR on D3. Transcript levels of alveolar epithelial cell type 1 (*Aqp5*) and 2 (*Sftpc*) markers were found to be significantly increased in *CreERT2* BLM-challenged lungs. However, IGF1R-deficient lungs demonstrated significantly decreased levels of the hypoxia-inducible factor 1 subunit alfa (*Hif1a*) (Fig. 8a). Since the RNA-seq indicated that the anti-oxidative stress marker *Gpx8* was the most up-regulated gene in *CreERT2* unchallenged lungs, its mRNA expression was assayed in BLM-treated lungs, and also found to be increased in IGF1R-deficient mice with respect to controls (Fig. 8a). SFTPC and HIF1A expression determined by immunohistochemistry verified mRNA expression levels (Fig. 8b). In accordance, the number of SFTPC^+^ cells was significantly increased (1.6-fold) (Fig. 8c), and conversely, HIF1A relative fluorescence intensity was found to be diminished (1.8-fold) in IGF1R-deficient lungs (Fig. 8d).

## Discussion

This is the first report of the functional implication of IGF1R in acute lung inflammation using a BLM mouse model. First, we analyzed the lung transcriptome in recently reported IGF1R-deficient mice (*CreERT2*)^23^ identifying differentially expressed genes with potentially protective roles. After BLM challenge, *CreERT2* mice showed resistance to BLM-mediated acute lung injury by counteracting lung inflammation and alveolar damage.

Lung transcriptome analysis of *CreERT2* mice demonstrated a general inhibition of differentially expressed genes, as similarly reported in prenatal *Igf1*-deficient lungs^24^. H1 histones *Hist1h1d* and *Hist1h2bb*, as well as *Hist1h4m* and *Hist1h1a* (Supplementary Table S1) were found to be up-regulated in *CreERT2* lungs. In this regard, it is widely known that H1 histones participate in chromatin condensation therefore repressing gene expression^25, 26^. Additionally, the histone acetyltransferases *Crebbp* and *Ep300*, both transcriptional co-activators, were found down-regulated in IGF1R-deficient lungs. In an inflammatory context, CREBBP and EP300 were reported to activate NF-κB-mediated pro-inflammatory gene expression in response to oxidative stress^26–28^. Thus, increased H1 histone together with lower acetyltransferase expression would result in a more condensed chromatin state, less accessible to transcription factors. Furthermore, the lower expression observed for mitochondrial respiratory chain complexes I (*mt-Nd4*, *mt-Nd5* and *mt-Nd6*) and III (*mt-Cytb*) genes (Supplementary Table S1) could result in decreased electron transport chain activity and consequently, in a reduction of reactive oxygen species production, since these complexes were reported to govern the response to hypoxia^29, 30^. In parallel, *Gpx8* and *Cyp1a1*, both involved in alleviating oxidative stress and inflammation^31, 32^, were the two most up-regulated genes. Overall, these results indicate that IGF1R deficiency could potentially be associated with a higher capacity to endure oxidant-induced injury.

After BLM treatment *CreERT2* mice showed improved survival. Similar results were observed in acute lung injury mouse models with compromised IGF1R activity^20, 33^. Moreover, IGF1R-deficient lungs showed increased *Igfbp3*, *Igfbp5*, *Insr* and *Foxo1* levels after BLM challenge, possibly due to compensatory effects in response to IGF1R deficiency, as reported^18, 34^. Specifically, IGFBP3 and IGFBP5 have shown protective properties in the mouse lung^35–37^.

Remarkably, *CreERT2* mice showed decreased total proteins in BALF, an indicator of reduced vascular permeability. This finding together with diminished erythrocyte counts are in accordance with the lower vascular extravasation reported in hypomorphic IGF1R-deficient mice^20^. Furthermore, the decreased presence of different inflammatory cell types in *CreERT2* lungs was reflected in BALF cell counts, and supported by reduced proliferation in perivascular and alveolar areas. Diminished *Cxcl1* and *Ly6g* mRNA levels were verified by reduced neutrophilic infiltration into *CreERT2* lungs. Considering that normal neutrophil bone marrow counts in mice are around 36.9%^38^, BLM clearly induced bone marrow neutrophilopoiesis in *Igf1r* ^*fl*/*fl*^ (54.25%) mice, unlike in IGF1R-deficient mice (25.44%). Similarly, pharmacological IGF1R blocking was recently reported to decrease the number of peripheral white blood cells^17, 39^. Altogether, these results demonstrate that the lack of IGF1R efficiently counteracts the acute BLM-induced neutrophilia, a major inflammatory player in this model.

Concerning TNF and *Il1b*, *CreERT2* lungs showed decreased expression of these cytokines, the most relevant in the lung during the early phase of BLM response^10, 40^. Accordingly, PREX1, an IGF1R signalling activator, has been shown to have a pro-inflammatory role after BLM treatment, and *Prex1-*deficient mice mirrored the pro-inflammatory profile shown by our IGF1R-deficient mice at D3^41^. In addition, activation of the IGF1R/PI3K/AKT/mTOR signalling pathway was reported to promote lung injury and repair^42, 43^, and IGF1R signaling promotes TNF-induced activation of NF-kB, a major pathway involved in inflammation^44^. Likewise, IGF1R plays an important role in initiation of the inflammatory process, as ablation of the macrophage IGF1/IGF1R signaling axis in mice inhibits the NLRP3 inflammasome, a protein complex triggered in the lung upon BLM-induced damage^21, 22^. During inflammation, while M1 macrophages contribute to tissue injury after excessive production of pro-inflammatory mediators (e.g., TNF and IL1B), M2 macrophages lead to resolution of inflammation and tissue repair upon anti-inflammatory cytokine activation (e.g., IL13 and CSF1)^9, 45^. In this regard, both diminished expression of *Tnf*, *Il1b* and *Il6* as well as elevated levels of *Csf1*, *Il13* and *Cd209a* found in *CreERT2* lungs would promote a pulmonary environment enriched in M2 macrophages. Noteworthy, IL13 was reported to protect against acute hyperoxic lung injury and *Cd209a* expression was found to be increased in the resolution-phase macrophages after peritonitis induction in mice^46, 47^. Altogether, these data support the idea that IGF1R deficiency would facilitate dampening of innate/adaptive immunity and resolution of inflammation.

Following BLM-induced lung injury we found increased expression of the alveolar markers *Aqp5* and SFTPC in *CreERT2* lungs. Accordingly, AQP5 expression was reported to be decreased in BLM-challenged lungs^48^, and its reduced levels were shown to contribute to abnormal fluid fluxes during pulmonary inflammation in mice^49^. In addition, SFTPC-deficient mice had increased mouse mortality, neutrophilic inflammation, and alveolar damage following BLM treatment^50^. In line with our results, *Sfptc* mRNA levels were also found to be increased in *CreERT2* lungs after allergic airway inflammation^51^. Thus, it appears that IGF1R deficiency confers a protective role against alveolar damage.

As a master transcriptional regulator of the adaptive response to hypoxia, HIF1A uses CREBBP and EP300 as transcriptional co-activators. Thus, decreased *Crebbp* and *Ep300* transcriptional levels in non-challenged *CreERT2* lungs could contribute to HIF1A reduced expression after BLM challenge. In accordance, alveolar type 2 cell-specific *Hif1a* knockout mice demonstrated milder pulmonary inflammation^52^, supporting that HIF1A could play an important role in acute lung inflammation.

Although we demonstrate a significant reduction of IGF1R expression in *CreERT2* lungs, TMX-mediated IGF1R deletion may occur with different degrees of mosaicism in different cell types. Thus, IGF1R generalized deletion cannot be used to deduce in which cells IGF1R signaling is crucial for promoting acute lung inflammation. Furthermore, the variability of intratracheal administration of BLM and the effect of the genetic background on phenotypic variations should also be considered as constraints to this report.

In summary, we have shown that IGF1R deficiency in mice plays a key role in decreasing transcriptional activity, and confers protection against alveolar damage and pulmonary inflammation. Notably, our findings may contribute to understanding the importance of IGF1R as a potential target for future therapeutic approaches in respiratory diseases with persistent damage and inflammation.

## Methods

### Ethics Statement

All experiments and animal procedures were carried out following the guidelines laid down by the European Communities Council Directive of 24 November 1986 (86/609/EEC) and were revised and approved by the CIBIR Bioethics Committee (refs 03/12 and 13/12). All animals were bred and maintained under specific pathogen-free (SPF) conditions in laminar flow caging at the CIBIR animal facility.

### Generation of *Igf1r*-deficient mice, establishment of the BLM-induced acute lung injury murine model and survival rate

*UBC-Cre-ERT2*; *Igf1r* ^*fl*/*fl*^ double transgenic mice were in a C57BL/6 enriched (at least six generation backcrosses to C57BL/6 strain) mixed genetic background. For experimental purposes, *UBC-Cre-ERT2*; *Igf1r* ^*fl*/*fl*^ double transgenic mice were crossed with *Igf1r* ^*fl*/*fl*^ mice to directly generate descendants in equal proportions in the same litter, and *Igf1r* ^*fl*/*fl*^ and *UBC-Cre-ERT2*; *Igf1r* ^*fl*/*fl*^ littermates were respectively used as experimental controls and mutants. Tamoxifen (TMX) was administered daily for five consecutive days to four-week-old mice of both genotypes to induce a postnatal *Igf1r* gene conditional deletion in *UBC-Cre-ERT2*; *Igf1r* ^*fl*/*fl*^ mice, as previously described^23^. Two-month-old and six-week-old tamoxifen-treated *UBC-CreERT2*; *Igf1r* ^*fl*/*fl*^ (*CreERT2*) and *Igf1r* ^*fl*/*fl*^ mice were used for RNAseq analysis, and BLM treatment to induce lung injury, respectively. Six-week-old mice (equal sex proportions) of both genotypes were intra-tracheally instilled with either a single dose of 2.5 µl/g body weight of BLM sulfate (5 U/kg) (EMD Millipore, Billerica, MA) in saline (2 U/ml) or saline (SAL) at D0, under a ketamine-xylazine anesthetic combination in saline (100:10 mg/kg respectively; 10 μ﻿l﻿﻿/g). Animals were monitored for 21 days to determine survival rates of both *UBC-CreERT2*; *Igf1r* ^*fl*/*fl*^ and *Igf1r* ^*fl*/*fl*^ mice after BLM challenge. Those animals that reached the human endpoint, as specified by the CIBIR Bioethics Committee protocol ref. 13/12, were also considered to be dead animals at each time point. The human endpoint criteria were applied when there was severe involvement of one of the specific (body weight, respiratory pattern and bleeding), or moderate involvement of two or more of the general (appearance, natural behavior) or specific parameters occurred, according to our expert veterinary evaluation. For acute lung injury studies, animals were sacrificed and tissues were collected at D3 (Fig. 2a).

### Tissue and BALF collection

Before tissue collection, animals were euthanized by intraperitoneal injection of 10 μ﻿l﻿/g of﻿ a ketamine-xylazine anesthetic combination in saline (300:30 mg/kg respectively). Three different sets of mice were used for BALF, mRNA/Western blot/histology and ELISA, respectively. A first set was used to obtain BALF; lungs from saline or BLM-treated mice were lavaged twice with 0.8 ml cold PBS. From the second set, left lungs were inflated with formalin fixative, post-fixed by immersion in formalin for 8–10 h, embedded in paraffin and cut into 3 µm sections for histopathological evaluation or immunohistochemistry; and right lobes were separated and snap frozen in liquid nitrogen for qRT-PCR and Western blot. Left lungs from a third set of animals were harvested for ELISA analysis.

### Quantification of BALF

Total cell number was counted and expressed as cells/ml BALF and differential cell counts were performed on May-Grünwald/Giemsa (Sigma-Aldrich, St. Louis, MO) stained cytospins from 4 animals per condition, blind counting a minimum of 300 cells per slide. Cells were determined to be macrophages, lymphocytes and neutrophils using standard morphology criteria. The average number of red blood cells per high-power field was obtained by evaluating 5 different fields on BALF cytospin preparations. Total protein concentration in BALF supernatants was determined using the Pierce BCA Protein Assay Kit (Thermo Fisher Scientific, Waltham, MA).

### Histopathological analyses and immunostaining

Quantification of inflammation was determined in H&E stained sections and expressed as the percentage of inflamed lung area to total section surface. Five BLM-treated left lungs per genotype were used. Inflamed lung areas were defined as darker H&E stained foci, where inflammatory cells accumulate massively, and delimitated manually using the Fiji ﻿open-source image processing software package v1.48r (https://fiji.sc).

Immunohistological detection of proliferating cells was performed as described^23^. Ki67 positive cells were counted using 5 or 10 fields per section per animal in perivascular and alveolar areas, respectively. The Ki67-labeling index was calculated as the number of Ki67 positive cells compared to the total cell number. Other histological and immunohistochemical quantifications were performed using 5 BLM-treated animals per genotype evaluating 5 different fields per lung. Determination of neutrophil and lymphocyte infiltration grades were assessed in lung perivascular areas on H&E and CD3 (ab5690 1:200, Abcam, Cambridge, UK) stained lung sections, and expressed as the number of neutrophils or lymphocytes to total cell infiltrates. Quantification of macrophages and alveolar type 2 cells was assessed in alveolar areas immunostained with anti-F4/80 (Clone MCA497GA 1:100, Bio-Rad, Langford, UK), and anti-SFTPC (AB3786, EMD Millipore, Billerica, MA) antibodies respectively, and expressed per unit area. Macrophage diameters were measured and volumes were extrapolated using the sphere volume formula. HIF1A expression was determined using the HIF1A antibody (ab2185 1:100, Abcam, Cambridge, UK) and expressed as relative fluorescence units (RFU) as previously described^18^. HIF1A relative fluorescence was evaluated using the Fiji software package.

### Femoral bone marrow isolation

Bone marrow (BM) isolation was performed using 5 BLMtreated animals per genotype (1 femur per animal). After dissection, the femoral heads were incised and femurs were positioned in bottom perforated 0.5 ml tubes placed inside 1.5 ml tubes. After centrifugation at 10000 × g for 15 seconds, BM was suspended in 500 µl PBS and centrifuged at 300 × g for 5 min at 4 °C. Following aspiration of the supernatant, BM pellets were resuspended in 500 µl of ACK lysing buffer (Thermo Fisher Scientific, Waltham, MA) and after 10 minutes of incubation, 1 ml of PBS was added. Total cell numbers were counted and expressed as cells/ml BM and neutrophil counts were performed on May-Grünwald/Giemsa (Sigma-Aldrich, St. Louis, MO) stained cytospins and 5 different fields per slide were blind counted.

### RNA isolation, reverse transcription and qRT-PCR

Inferior lung lobes taken from eight-week-old mice or on D3 post-bleomycin were homogenized in TRIzol Reagent (Invitrogen, Carlsbad, CA) and total RNA was isolated using an RNeasy Mini Kit (Qiagen, Hilden, Germany). RNA from D3 post-bleomycin-treated mice was reverse transcribed to cDNA using SuperScript II First-Strand Synthesis System (Invitrogen, Carlsbad, CA) as per the manufacturer’s specifications. cDNA samples were amplified by qRT-PCR in triplicate on a 7300 Real Time PCR instrument (Applied Biosystems, Foster City, CA), for each primer pair assayed (Supplementary Table S4). Results were normalized using the 18S rRNA gene as the endogenous control.

### Lung transcriptome analysis

RNAseq analysis was performed on eight-week-old tamoxifen-treated *UBC-CreERT2*; *Igf1r* ^*fl*/*fl*^ and *Igf1r* ^*fl*/*fl*^ lungs (n = 3 per genotype). Approximately, 1 μg of total RNA from each sample was submitted to the CIBIR Genomics Core Facility for sequencing. Briefly, after verifying RNA quality in an Experion Bioanalyzer (BioRad), TruSeq total RNA libraries were generated according to the manufacturer’s instructions (Illumina Inc). The libraries were sequenced in a Genome Analyzer IIx (Illumina Inc) to generate 150 single-end reads. *Mus musculus* GRCm38.71 (FASTA) from the Ensemble database was used as the reference genome. After removing adapter sequences with the Cutadapt software^53^, mapping to the reference genome was performed with TopHat2 (version 2.5)^54^. Gene expression quantification, normalization, and statistical analyses were performed with SeqSolve (Integromics). Expression data were normalized by calculating the fragments per kilobase of exon per million fragments mapped (FPKM) reads for each gene. *Igf1r*-transcriptionally regulated genes involved in biological processes were classified according to GO and Keyword annotations. Additionally, a PubMed search, and the Genecards and OMIM tools were used to help assign biological functions.

### Western blot analysis

Superior lung lobes were solubilized in a 10 mM Tris/HCl (pH 7.4) buffer containing 0.1% sodium dodecyl sulfate, a protease inhibitor mixture, and DNase (Promega, Fitchburg, WI). Samples were separated in NuPAGE Novex 4–12% Bis-Tris Gel (Invitrogen, Carlsbad, CA) and transferred to a polyvinylidene difluoride membrane (EMD Millipore). Membranes were incubated with primary antibodies for IGF1R (#3027 Cell Signalling Inc., Danvers, MA) and beta-Actin (ab6276 Abcam, Cambridge, UK) at 1:1000 and 1:30000 dilutions respectively, and then incubated with horseradish peroxidase-conjugated anti-rabbit or anti-mouse antibodies (DAKO, Agilent technologies, Santa Clara, CA) for IGF1R and beta-Actin respectively, at a 3:10 dilution. Signals were detected using ECL Western Blot Substrate (Thermo Fisher Scientific, Waltham, MA) and Hyperfilm ECL (GE Healthcare, Little Chalfont, UK). Films were scanned and signals in the linear range were quantified using Image J and normalized to beta-Actin levels.

### ELISAS

TNF and IL13 levels were determined in homogenized tissue lysates using left lungs with the help of mouse TNF-alpha Quantikine and IL-13 Duoset ELISA Kits (R&D Systems, Minneapolis, MN), according to the manufacturer’s guidelines.

### Statistics

Statistical analyses were performed using SPSS Statistics Software v21 for Windows. Differences between genotypes were evaluated for significance using the non-parametric Mann-Whitney U test or the Dunn Sidak test for multiple comparisons. Results are shown as mean values ± standard error of the mean (SEM). For all analyses, a *p* value < 0.05 was considered statistically significant.

## Electronic supplementary material

Supplementary information
