# Supplementary material for: IGF1R deficiency attenuates acute inflammatory response in a bleomycin-induced lung injury mouse model
Source: Sci Rep. 2017 Jun 27;7:4290. doi: 10.1038/s41598-017-04561-4 (PMC5487362; doi:10.1038/s41598-017-04561-4)
Supplement: Supplementary file 1 — Supplementary information [file 41598_2017_4561_MOESM1_ESM.pdf]

Supplementary Information for

Igf1r deficiency attenuates acute inflammatory response in a bleomycin-induced lung injury mouse model

Sergio Piñeiro-Hermida[1], Icíar P. López[1], Elvira Alfaro-Arnedo[1], Raquel Torrens[1], María Iñiguez[2], Lydia Alvarez-Erviti[3], Carlos Ruíz-Martínez[4], and José G. Pichel [1,*]

[1] Lung Cancer and Respiratory Diseases Unit, [2] Genomics Core Facility and [3] Molecular Neurobiology Unit; Centro de Investigación Biomédica de la Rioja (CIBIR), Fundación Rioja Salud. [4] Pneumology Service, Hospital San Pedro. Logroño, Spain.

Files included:

Supplementary Table S1. Differential gene expression determined by RNAseq in the lung of *UBC-CreERT2; Igf1r*$^{fl/fl}$ mutant mice (FDR < 0.2).

Supplementary Table S2. Biological functions and differentially expressed genes found with significant changes after RNAseq in the lung of *UBC-CreERT2; Igf1r*$^{fl/fl}$ mutant mice (FDR < 0.1).

Supplementary Table S3. Differential BALF cell counts represented as the percentage of each cell type to total cell counts in *Igf1r*$^{fl/fl}$ and *UBC-CreERT2; Igf1r*$^{fl/fl}$ mice.

Supplementary Table S4. Primer sets used for qRT-PCR .

Supplementary Table S1. Differential gene expression determined by RNAseq in the lung of *UBC-CreERT2; Igf1r*$^{fl/fl}$ mutant mice (FDR < 0.2)

Position	Gene name	Description	Log2 fold change	Q-value/FDR
**1**	Gpx8	Glutathione peroxidase 8	33,33	5,08097E-11
**2**	Igf1r	Insulin-like growth factor I receptor	-2,35512	6,91951E-11
**3**	Cyp1a1	Cytochrome P450, family 1, subfamily a, polypeptide 1	2,22399	1,51895E-10
**4**	Gnptg	N-acetylglucosamine-1-phosphotransferase, gamma subunit	33,33	1,10271E-08
**5**	Srrm2	Serine/arginine repetitive matrix 2	-1,29438	2,90396E-06
**6**	Nr1d2	Nuclear receptor subfamily 1, group D, member 2	-1,43483	2,65234E-05
**7**	Ppp1r2-ps4	Protein phosphatase 1, regulatory (inhibitor) subunit 2, pseudogene 4	33,33	3,69653E-05
**8**	Saa3	Serum amyloid A 3	2,58211	7,26024E-05
**9**	Epas1	Endothelial PAS domain protein 1	-1,17128	8,78217E-05
**10**	Spon2	Spondin 2, extracellular matrix protein	1,76138	0,000320111
**11**	Slco2a1	Solute carrier organic anion transporter family, member 2a1	-1,08642	0,000596572
**12**	Mll2	Lysine (K)-specific methyltransferase 2D	-1,11086	0,000614606
**13**	Npnt	Nephronectin	-0,950843	0,000614606
**14**	Hydin	HYDIN, axonemal central pair apparatus protein	-1,51408	0,00123442
**15**	Pcdh1	Protocadherin 1	-0,92639	0,00123442
**16**	Lyz1	Lysozyme 1	1,00162	0,00130818
**17**	Top2a	Topoisomerase (DNA) II alpha	1,19316	0,00146147
**18**	Zbtb34	Zinc finger and BTB domain containing 34	-33,33	0,00169479
**19**	Cyyr1	Cysteine and tyrosine-rich protein 1	-0,864652	0,00169479
**20**	mt-Nd5	NADH dehydrogenase 5, mitochondrial	-0,946779	0,00436231
**21**	Ces1g	Carboxylesterase 1G	2,10737	0,00454152
**22**	Pcnx	Pecanex homolog (Drosophila)	-0,989945	0,00483008
**23**	Muc5b	Mucin 5, subtype B, tracheobronchial	-1,04542	0,0066965
**24**	Med13l	Mediator complex subunit 13-like	-1,05975	0,0072912
**25**	Lox	Lysyl oxidase	-0,893177	0,00758989
**26**	Tenc1	Tensin 2	-0,95569	0,0130699
**27**	Dnahc6	Dynein, axonemal, heavy chain 6	-0,953513	0,0156303
**28**	Hist1h1d	Histone cluster 1, H1d	1,27832	0,0174382
**29**	Atp2a3	ATPase, Ca++ transporting, ubiquitous	-0,935091	0,0195047
**30**	Zbtb16	Zinc finger and BTB domain containing 16	-0,87579	0,0255043
**31**	Svep1	Sushi, von Willebrand factor type A, EGF and pentraxin domain containing 1	-0,82965	0,0288283
**32**	Mki67	Antigen identified by monoclonal antibody Ki 67	0,88978	0,0288283
**33**	Snhg11	Small nucleolar RNA host gene 11	-1,27045	0,0331612
**34**	Hist1h2bb	Histone cluster 1, H2bb	1,21471	0,0347587
**35**	Zfp518b	Zinc finger protein 518b	-33,33	0,0360491
**36**	Zfhx3	Zinc finger homeobox 3	-0,987494	0,0371726
**37**	Notch3	Neurogenic locus homolog protein 3	-1,01305	0,0405676
**38**	Foxo1	Forkhead box O1	-1,02458	0,0411093
**39**	Apol11b	Apolipoprotein L 11b	-1,89341	0,0485738
**40**	Syngap1	Synaptic Ras GTPase activating protein 1 homolog (rat)	-33,33	0,0487062
**41**	AC147987.1	RIKEN cDNA 4933404O12 gene	33,33	0,0492756
**42**	C030017K20Rik	RIKEN cDNA C030017K20 gene	-33,33	0,0507919
**43**	Rab6b	RAB6B, member RAS oncogene family	-1,17859	0,0507919
**44**	Crebbp	CREB binding protein	-0,854579	0,0508175
**45**	Kif11	Kinesin family member 11	1,18653	0,0508175
**46**	Fancf	Fanconi anemia, complementation group F	33,33	0,0536387
**47**	Hr	Hairless	-33,33	0,0546633
**48**	Ptprb	Protein tyrosine phosphatase, receptor type, B	-0,815991	0,0550762
**49**	Gdpd3	Glycerophosphodiester phosphodiesterase domain containing 3	-1,6275	0,0593667
**50**	Marco	Macrophage receptor with collagenous structure	1,43551	0,0632713
**51**	Tet3	Tet methylcytosine dioxygenase 3	-0,969638	0,065462
**52**	mt-Cytb	Cytochrome b, mitochondrial	-0,768723	0,0674572
**53**	Luc7l2	LUC7-like 2 (S. cerevisiae)	-0,472481	0,0674572
**54**	Nav2	Neuron navigator 2	-0,969168	0,0684272
**55**	Ppp1r3c	Protein phosphatase 1, regulatory (inhibitor) subunit 3C	1,13441	0,0712999
**56**	Trim25	Tripartite motif-containing 25	-1,04788	0,0733889
**57**	Prrc2c	Proline-rich coiled-coil 2C	-0,785804	0,0733889
**58**	Mlxip	MLX interacting protein	-0,877622	0,076686
**59**	Polr2a	Polymerase (RNA) II (DNA directed) polypeptide A	-0,816322	0,0776129
**60**	Ep300	E1A binding protein p300	-0,79195	0,0777767
**61**	Abi3bp	ABI gene family, member 3 (NESH) binding protein	-0,842908	0,0783232
**62**	Tgfbr3	Transforming growth factor, beta receptor III	-0,794265	0,0902875
**63**	Efnb2	Ephrin B2	-0,983599	0,0926999
**64**	Hnrnpul2	Heterogeneous nuclear ribonucleoprotein U-like 2	-0,81716	0,0936172
**65**	Rps7	Ribosomal protein S7	1,53422	0,093661
**Genes with FDR between 0.10 and 0.20**				
**66**	Cd5l	CD5 antigen-like	2,38007	0,11316
**67**	Hist1h4m	Histone cluster 1, H4m	33,33	0,114229
**68**	Pdzd2	PDZ domain containing 2	-0,725257	0,115581
**69**	Btbd3	BTB (POZ) domain containing 3	-0,70643	0,119155
**70**	Sorbs3	Sorbin and SH3 domain containing 3	-0,908928	0,120407
**71**	Myo1d	Myosin ID	-0,711134	0,120505
**72**	Tubd1	Tubulin, delta 1	33,33	0,124497
**73**	Ska1	Spindle and kinetochore associated complex subunit 1	33,33	0,124497
**74**	Fam65a	Family with sequence similarity 65, member A	-1,0518	0,124918
**75**	Dnahc1	Dynein, axonemal, heavy chain 1	-1,39517	0,127394
**76**	mt-Nd6	NADH dehydrogenase 6, mitochondrial	-0,805532	0,127994
**77**	Pom121	Nuclear pore membrane protein 121	33,33	0,127994
**78**	Bmpr2	Bone morphogenetic protein receptor, type II (serine/threonine kinase)	-0,708527	0,129371
**79**	Hist1h1a	Histone cluster 1, H1a	1,21029	0,129371
**80**	Ptprg	Protein tyrosine phosphatase, receptor type, G	-0,688689	0,129504
**81**	Eif2c2	Argonaute RISC catalytic subunit 2	-0,799781	0,134313
**82**	Arhgap31	Rho GTPase activating protein 31	-0,642048	0,134313
**83**	Igfbp3	Insulin-like growth factor binding protein 3	0,800809	0,158183
**84**	D730039F16Rik	CutA divalent cation tolerance homolog-like	33,33	0,163078
**85**	Pkd1	Polycystic kidney disease 1 homolog	-0,696951	0,16421
**86**	Baz2a	Bromodomain adjacent to zinc finger domain, 2A	-0,812446	0,168588
**87**	Atp5k	ATP synthase, H+ transporting, mitochondrial F1F0 complex, subunit E	1,02612	0,169495
**88**	Cd276	CD276 antigen	33,33	0,172103
**89**	Gm10277	Predicted gene 10277	-33,33	0,174175
**90**	Dock6	Dedicator of cytokinesis 6	-0,848506	0,175012
**91**	Parp4	Poly (ADP-ribose) polymerase family, member 4	-0,838119	0,175012
**92**	Plxna2	Plexin A2	-0,703027	0,175012
**93**	Sdf2l1	Stromal cell-derived factor 2-like 1	1,20602	0,178254
**94**	Nsun5	NOL1/NOP2/Sun domain family, member 5	33,33	0,178254
**95**	Crtc3	CREB regulated transcription coactivator 3	-1,0953	0,182596
**96**	Sh3pxd2a	SH3 and PX domains 2A	-0,669411	0,182596
**97**	Arid1a	AT rich interactive domain 1A (SWI-like)	-0,819629	0,182803
**98**	Crim1	Cysteine rich transmembrane BMP regulator 1 (chordin like)	-0,714825	0,185107
**99**	Smarca1	SWI/SNF related, matrix associated, actin dependent regulator of chromatin, subfamily a, member 1	33,33	0,18851
**100**	2900026A02Rik	RIKEN cDNA 2900026A02 gene	-1,04303	0,191335
**101**	Fmnl2	Formin-like 2	-0,82042	0,191335
**102**	Shank2	SH3/ankyrin domain gene 2	-1,65139	0,193573
**103**	Pabpc1	Poly(A) binding protein, cytoplasmic 1	-0,800107	0,193573
**104**	mt-Nd4	NADH dehydrogenase 4, mitochondrial	-0,676625	0,197356
**105**	Brd4	Bromodomain containing 4	-0,965817	0,198125
**106**	Hspg2	Perlecan (heparan sulfate proteoglycan 2)	-0,768995	0,198125
**107**	Reck	Reversion-inducing-cysteine-rich protein with kazal motifs	-0,854115	0,198715

Supplementary Table S2. Biological functions and differentially expressed genes found with significant changes after RNAseq in the lung of *UBC-CreERT2; Igf1r*$^{fl/fl}$ mutant mice (FDR < 0.1).

Biological functions	Up-regulated genes	Down-regulated genes
Development and growth	*Saa3, Mki67, Kif11, Fancf, Ppp1r3c, Rps7*	*Epas1, Npnt, Pcdh1, Lox, Tenc1, Zbtb16, Zfhx3, Notch3, Foxo1, Apol11b, Syngap1, Crebbp, Prprb, Luc7l2, Nav2, Prrc2c, Ep300, Abi3bp, Tgfbr3, Efnb2*
Transcriptional regulation	*Hist1h1d, Hist1h2bb, Ppp1r3c, Rps7*	*Srrm2, Nr1d2, Epas1, Zbtb34, Med13l, Zbtb16, Zfp518b, Zfhx3, Notch3, Foxo1, Crebbp, Hr, Tet3, Luc7l2, Mlxip, Polr2a, Ep300, Hnrnpul2*
Epigenetics	*Top2a, Hist1h1d, Hist1h2bb, Fancf*	*Mll2, Foxo1, Rab6b, Crebbp, Tet3, Polr2a, Ep300*
Inflammation and Immune response	*Saa3, Spon2, Lyz1, Marco*	*Nr1d2, Slco2a1, Muc5b, Foxo1, Trim25, Tgfbr3*
Protumoral activity	*Tp2a, Mki67*	*Epas1, Mll2, Atp2a3, Notch3, Trim25, Prrc2c, Abi3bp, Efnb2*
Hypoxia, redox and oxidative stress	*Gpx8, Cyp1a1*	*Nr1d2, Epas1, mt-Nd5, Foxo1, Hr, mt-Cytb, Ep300, Tgfbr3*
Cell adhesion and migration	*Spon2*	*Npnt, Pcdh1, Tenc1, Svep1, Nav2, Tgfbr3, Efnb2*
Tumor suppression	-	*Zbtb16, Lox, Zfhx3, Notch3, Foxo1, Ep300, Tgfbr3*
Respiratory diseases	*Saa3, Marco*	*Epas1, Pcdh1, Muc5b, Notch3*
Vascular permeability and Hypertension	*Spon2*	*Epas1, Lox, Foxo1, Ptprb*
Insulin regulation and resistance	*Saa3*	*Epas1, Mll2, Atp2a3, Foxo1*
Metabolism	*Ces1g, Ppp1r3c*	*Nr1d2, Foxo1, Gdpd3*
Regulated by Igf action and signalling	-	*Muc5b, Lox, Foxo1, Crebbp, Efnb2*
Detoxification and damage resistance	*Cyp1a1, Spon2, Ces1g*	*Epas1, Notch3*
Cell transport	-	*Atp2a3, Apol11b, Rab6b*
Ciliary motility	*Kif11*	*Hydin, Dnahc6*
Other/Unknown	*Gnptg, Ppp1r2-ps4, Ac147987.1*	*Cyyr1, Pcnx, Snhg11, C030017K20Rik, Syngap1*

Supplementary Table S3. Differential BALF cell counts represented as the percentage of each cell type to total cell counts in *Igf1r*$^{fl/fl}$ and *UBC-CreERT2; Igf1r*$^{fl/fl}$ mice.

**Cell type**	**Condition**	***Igf1r***$^{fl/fl}$	***CreERT2***
Neutrophils	SAL	0.833 ± 0.166	0.833 ± 0.396
	BLM	55.416 ± 1.535	17.250 ± 3.119 ***
Macrophages	SAL	97.583 ± 0.567	98.998 ± 0.192
	BLM	37.166 ± 2.558	73.250 ± 2.495 ***
Lymphocytes	SAL	1.583 ± 0.416	0.999 ± 0.360
	BLM	8.333 ± 0.990	9.500 ± 1.040

Data are expressed as mean ± SEM. *** $p < 0.001$, when comparing *Igf1r*$^{fl/fl}$ *vs. UBC-CreERT2; Igf1r*$^{fl/fl}$ on each condition. SAL, saline; BLM, bleomycin.

Supplementary Table S4. Primer sets used for qRT-PCR.

Gene	Accession No.	Forward primer (5´-3´)	Reverse primer (5´-3´)
Adgre1	NM_010130.4	ATACCCTCCAGCACATCCAG	CTCCCATCCTCCACATCAGT
Aqp5	NM_009701	GGTGGTCATGAATCGGTTCAGC	GTCCTCCTCTGGCTCATATGTG
Cd209a	NM_133238.5	GAGATGACGGCTGGAATGAC	AGATGGTGGAGGGAGTTGG
Csf1	NM_007778.4	CGAGTCAACAGAGCAACCAA	TGCTTCCTGGGTCAAAAATC
Cxcl1	NM_008176.3	ATCCAGAGCTTGAAGGTGTTG	GTCTGTCTTCTTTCTCCGTTACTT
Foxo1	NM_019739.3	TTCTCTCGTCCCCAACATCT	TGCTGTCCTGAAGTGTCTGC
Gpx8	NM_027127.2	ACATTCCCCATCTTCCACAA	ATTCCACCTTGGCTCCTTCT
Hif1a	NM_010431.2	TTGGAACTGGTGGAAAAACTG	ACTTGGAGGGCTTGGAGAAT
Igf1	NM_010512	CAGAAGCGATGGGGAAAAT	GTGAAGGTGAGCAAGCAGAG
Igf1r	NM_010513	ATGGCTTCGTTATCCACGAC	AATGGCGGATCTTCACGTAG
Igfbp3	NM_008343.2	GCCCTCTGCCTTCTTGATTT	TCACTCGGTTATGGGTTTCC
Igfbp5	NM_010518.2	GATGAGACAGGAATCCGAACAAG	AATCCT TTGCGGTCACAGTTG
Il1b	NM_008361.3	GCAACTGTTCCTGAACTCAACT	ATCTTTTGGGGTCCGTCAACT
Il6	NM_031168.1	ACGGCCTTCCCTACTTCACA	CATTTCCACGATTTCCCAGA
Il13	NM_008355.3	GCCTCCCCGATACCAAAAT	CTTCCTCCTCAACCCTCCTC
Insr	NM_010568.2	TCCTGAAGGAGCTGGAGGAGT	CTTTCGGGATGGCCTGG
Ly6g	NM_001310438.1	CCTGGTTTCAGTCCTTCTGC	CACACACTACCCCCAACTCA
Marco	NM_010766.2	TCCCTGTGATGGAGACCTTC	GTGAGCAGGATCAGGTGGAT
Rn18s	NR_003278.3	ATGCTCTTAGCTGAGTGTCCCG	ATTCCTAGCTGCGGTATCCAGG
Sftpc	NM_011359	GAAGATGGCTCCAGAGAGCATC	GGACTCGGAACCAGTATCATGC
Tnf	NM_013693.3	GCCTCTTCTCATTCCTGCTTG	CTGATGAGAGGGAGGCCATT
